# Relationship between plasma S-Klotho and cardiometabolic risk in sedentary adults

**DOI:** 10.18632/aging.102771

**Published:** 2020-01-20

**Authors:** Francisco J. Amaro-Gahete, Lucas Jurado-Fasoli, Guillermo Sanchez-Delgado, José V. García-Lario, Manuel J. Castillo, Jonatan R. Ruiz

**Affiliations:** 1 EFFECTS-262 Research group, Department of Medical Physiology, School of Medicine, University of Granada, Granada, Spain; 2 PROmoting FITness and Health through Physical Activity Research Group (PROFITH), Sport and Health University Research Institute (iMUDS), Department of Physical and Sports Education, School of Sports Science, University of Granada, Granada, Spain; 3 Hospital Universitario Clínico San Cecilio, Granada, Spain

**Keywords:** insulin sensitivity, aging, cholesterol, glucose, biomarker

## Abstract

This cross-sectional study investigates the relationship between the shed form of the Klotho protein (S-Klotho) in plasma, and cardiometabolic risk in healthy, sedentary adults. The study subjects were 214 healthy, sedentary adults (~64% women). Data were collected during the baseline assessments of two randomized controlled trials: The FIT-AGEING study (n=74 [~50% women] middle-aged adults aged 40-65 years) and the ACTIBATE study (n=140 [~70% women] young adults aged 18-25 years). A sex-specific cardiometabolic risk score was calculated for each subject based on waist circumference, systolic and diastolic blood pressure, and plasma glucose, high-density lipoprotein cholesterol and triglycerides. A significant inverse relationship was detected between S-Klotho and the cardiometabolic risk score of both the middle-aged men and women (β=-0.658, R^2^=0.433, P<0.001 and β=-0.442, R^2^=0.195, P=0.007) which persisted after adjusting for actual age, energy intake, and VO_2_max. No significant association was found between S-Klotho and cardiometabolic risk score for the young, healthy adults (P>0.5), nor for the young, healthy men and women when analysed separately (all P>0.1). In conclusion, in healthy, sedentary, middle-aged adults, but not in young, healthy, sedentary adults, higher plasma S-Klotho concentrations are associated with a lower cardiometabolic risk score.

## INTRODUCTION

The shed form of the α-Klotho protein (S-Klotho) is thought to prevent some of the harmful consequences of ageing, increasing life expectancy when it is overexpressed but inducing premature ageing phenotypes when downregulated [1–3]. Studies that have investigated the physiological mechanisms behind the anti-ageing properties of S-Klotho report it to regulate mineral homeostasis, reduce intracellular oxidative stress, and attenuate chronic inflammation [1, 4, 5]. Its effect on longevity might therefore be explained, at least in part, via its metabolic functions.

Ageing is associated with an increased incidence of cardiometabolic disease [6] - the major cause of morbidity-mortality in developed countries [7]. The World Health Organization reports that 17.9 million people die every year due to cardiometabolic disease [8], and over a billion people around the world suffer its clinical consequences [9]. Evidence from clinical and experimental studies shows that oxidative stress and chronic inflammation are closely associated with cardiometabolic disorders such as obesity, type II diabetes mellitus and hypertension [10, 11]. Preserving the physiological functions that control intracellular oxidative stress and chronic inflammation might therefore help reduce the cardiometabolic risk associated with ageing [12].

Given the physiological functions of S-Klotho, and the pathophysiological mechanisms involved in the development of cardiometabolic disease, it is plausible that S-Klotho is cardioprotective. Little is known, however, about the relationship between S-Klotho and cardiometabolic risk in humans, although it has been reported that plasma S-Klotho is inversely associated with the prevalence of cardiometabolic disease in older adults [13]. Further, low S-Klotho plasma levels have been associated with the development of type II diabetes mellitus [14], an unhealthy body composition status [15], low physical fitness [16], and a higher risk of all-cause mortality [17].

Studying the ageing process in elderly populations suffers the limitation that the majority of subjects already have some form of ageing-related disease [18]. Thus, it is of clinical interest to investigate the physiological mechanisms operating in healthy, relatively young individuals who have no such ailments [19]. The present study, which investigates the association between plasma S-Klotho levels and cardiometabolic risk factors, involved two groups of healthy, sedentary adults aged 40-65 and 18-25 years.

## RESULTS

Supplementary Table 1 provides the descriptive characteristics of the study subjects.

**Figure 1:**
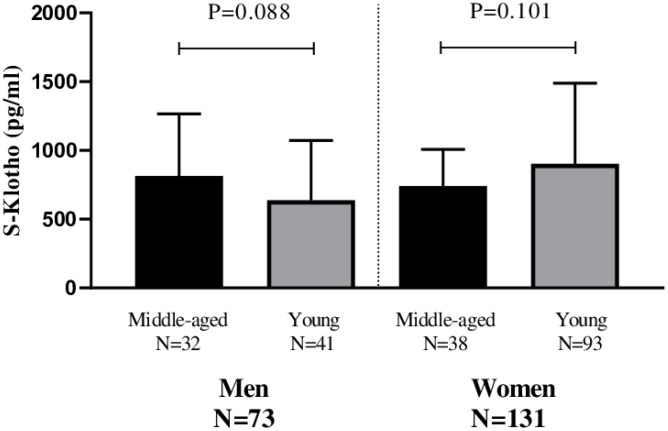
**Differences in plasma S-Klotho concentration between middle-aged and young men and women.**

No significant differences were seen in terms of plasma S-Klotho between the middle-aged and young men (P=0.088; Figure 1) and women (P=0.101; Figure 1)

**Figure 2:**
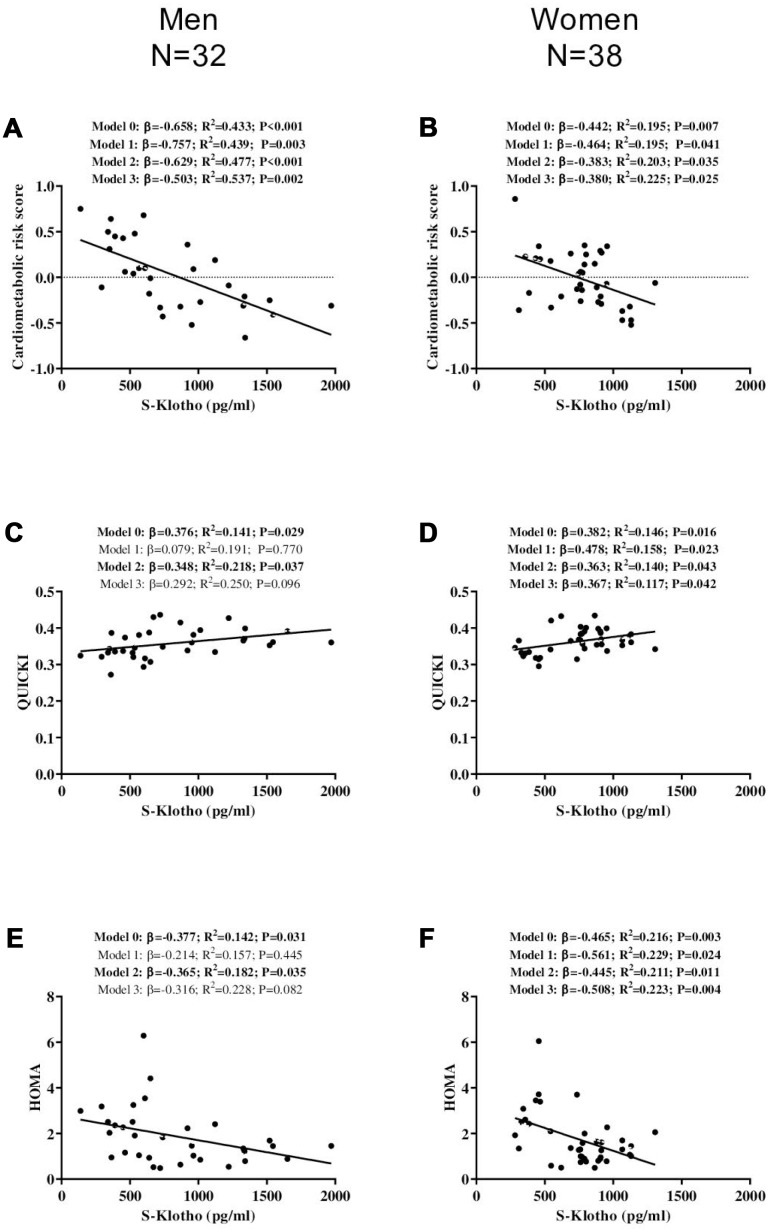
**Association between S-Klotho and the cardiometabolic risk index, the quantitative insulin sensitivity check index (QUICKI), and the homeostatic model assessment of insulin resistance index (HOMA) in middle-aged, sedentary adults.** β: standardized regression coefficient; R^2^ and P are provided for simple and multiple linear regression analyses. Model 0; unadjusted, Model 1; adjusted for age, Model 2; adjusted for energy intake, Model 3; adjusted for cardiorespiratory fitness.

A significant inverse association was detected between S-Klotho and the cardiometabolic risk score for the middle-aged men (*β*=-0.658, R^2^=0.433, P<0.001; Figure 2A) and women (*β*=-0.442, R^2^=0.195, P=0.007; Figure 2B); this persisted after adjusting for actual age, energy intake and VO_2_max (all P<0.05; Figure 2 panels 2A and 2B).

S-Klotho was positively related to QUICKI in the middle-aged men (*β*=0.376, R^2^=0.141, P=0.029; Figure 2C) and middle-aged women (*β*=0.382, R^2^=0.146, P=0.016 Figure 2D); this relationship persisted after adjusting for energy intake (P<0.05; Figure 2 panels 2C and 2D). In addition, these associations persisted in the middle-aged women after adjusting for age and VO_2_max (all P<0.05; Figure 2D), but disappeared in the middle-aged men (all P>0.09; Figure 2C).

A significant negative association was observed between S-Klotho and HOMA for both the both middle-aged men (*β*=-0.377, R^2^=0.142, P=0.031; Figure 2E) and women (*β*=-0.465, R^2^=0.216, P=0.003; Figure 2F), which persisted after adjusting for energy intake (all P<0.04; Figure 2H and Figure 2I). In addition, it persisted in the middle-aged women after controlling for actual age and VO_2_max (all P<0.03; Figure 2I), but not in the middle-aged men (all P>0.08; Figure 2 panels 2E and 2F).

**Figure 3:**
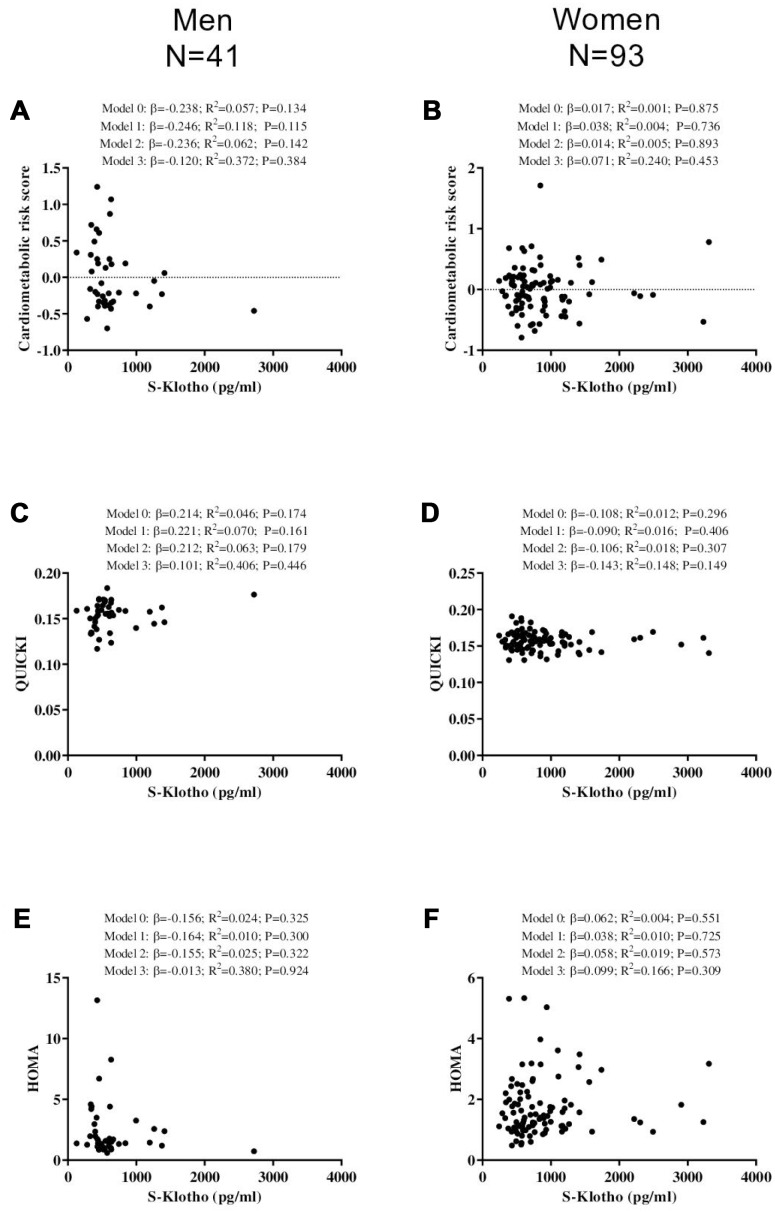
**Association between S-Klotho and the cardiometabolic risk index, the quantitative insulin sensitivity check index (QUICKI), and the homeostatic model assessment of insulin resistance index (HOMA) in young, sedentary adults.** β standardized regression coefficient; R^2^ and P are provided for simple and multiple linear regression analyses. Model 0; unadjusted, Model 1; adjusted for age, Model 2; adjusted for energy intake, Model 3; adjusted for cardiorespiratory fitness.

No significant association was seen between plasma S-Klotho and either cardiometabolic risk score, QUICKI or HOMA, in either the young men or young women (all P>0.134; Figure 3).

Supplementary Table 2 shows the associations between S-Klotho and blood pressure (systolic and diastolic), glycaemia, lipids, and liver function - unadjusted and controlling for several confounders.

None of the above-mentioned findings changed after adjusting for macronutrient intake (fat, carbohydrate, and protein intake), sedentary time and/or moderate-vigorous physical activity levels (data not shown).

## DISCUSSION

The present results show that plasma S-Klotho is inversely associated with cardiometabolic risk and insulin resistance in both sedentary middle-aged men and women (i.e. 40-65 years-old), independent of their actual age, cardiorespiratory fitness, physical activity levels, and dietary intake. No such associations were seen in the young adults (i.e. 18-25 years-old). These findings support the idea that S-Klotho provides a marker of cardiometabolic status in sedentary, middle-aged individuals.

Since the discovery of the α-Klotho gene [2], numerous studies have investigated the protein's genetic regulation and physiological functions. However, little work has been invested in determining whether plasma S-Klotho is related to cardiometabolic risk. Semba et al. [13, 17] reported a strong inverse association between S-Klotho and the likelihood of developing cardiovascular disease, as well as the risk of all-cause mortality, in a large cohort of adults aged over 65 years. Similarly, a recent study reports that patients with coronary artery disease have lower plasma S-Klotho concentrations, and show reduced α-Klotho gene expression in the vasculature walls [20]. Kitagawa et al. [21] observed that reductions in S-Klotho were independently associated with signs of vascular dysfunction, such as arterial stiffness, in patients with chronic kidney disease. Indeed, S-Klotho has been proposed a promising diagnostic biomarker and even a therapeutic agent for the treatment of cardiovascular disease [22]. However, Branderburg et al. [23] and Valenzuela et al. [24] reported S-Klotho not to be related to cardiovascular disease and all-cause mortality in a cohort of elderly persons with normal and mildly impaired kidney function, although they were at high risk of suffering a future cardiovascular event [23] and in elderly patients with chronic kidney disease [24], respectively. These discrepancies might be explained by the different health status and age of the participants involved in each study. To the best of our knowledge, no studies have examined the association of S-Klotho with cardiometabolic risk in healthy, sedentary young and middle-aged adults of both sexes. The present results suggest an inverse association exists between S-Klotho and cardiometabolic risk in individuals aged 40-65 years, supporting the idea that S-Klotho might also be a good indicator of cardiometabolic health in disease-free, middle-aged persons. However, no such associations were observed in the present young adults. These results are particularly surprising since the difference in the plasma S-Klotho levels of the middle-aged and young men and women only showed a trend towards significance; this might have a physiological (e.g., the better health status of young individuals) or methodological explanation (e.g., the blood samples of the FIT-AGEING and ACTIBATE projects were analysed at different times of the year). Clarification may come via further studies with larger samples. Finally, although we did not find statistical differences in S-Klotho levels between middle-aged and young adults cohorts, we observed similar S-Klotho levels than those reported by a previous study [25].

The cardiometabolic-protective effect of S-Klotho might be explained via its metabolic functions. S-Klotho attenuates vascular calcification and exerts a vasoprotective effect, increasing the production of nitric oxide via the upregulation of endothelial nitric oxide synthase activity [1], thus maintaining endothelial homeostasis [26]. S-Klotho is also a phosphaturic hormone that functions as a β-glucuronidase able to modify *NPT2A* gene expression and induce phosphaturia [27]. This leads to a reduction in plasma phosphate levels and also helps prevent vascular calcification [1]. Further, S-Klotho downregulates the production of pro-inflammatory cytokines [26]. Given that chronic inflammation is closely linked with the development of cardiometabolic diseases (e.g., metabolic syndrome and/or type II diabetes mellitus, among others) [28], this might reduce cardiometabolic risk.

There is considerable evidence showing that low S-Klotho is strongly associated with the development of type 2 diabetes mellitus in prediabetic patients [14], greater insulin resistance in patients with type 2 diabetes mellitus [29], and increased complications in such patients (e.g., diabetic nephropathy or diabetic coronary heart disease among others) [30, 31]. However, evidence is lacking on whether S-Klotho is linked to insulin sensitivity/resistance in healthy populations. The present results show that plasma S-Klotho is associated with insulin sensitivity in both healthy middle-aged men and women, independent of several confounding factors, and as such it is plausible that S-Klotho plays a role in regulating glycaemia and lipid metabolism. It has been reported that S-Klotho (i) downregulates the production of pro-inflammatory cytokines [26], and (ii) attenuates oxidative stress by increasing the forkhead box-O transcription factors that promote catalase and mitochondrial manganese-superoxide dismutase activity [32]. Since these physiological mechanisms help improve insulin sensitivity problems [33, 34], and given the associations found in the present study, S-Klotho might be considered a hormone that protects against the development of insulin resistance in healthy individuals aged 40-65 years.

The present work suffers from a number of potential limitations. Firstly, its cross-sectional design means no causal relationships can be established. Plasma S-Klotho might not sufficiently reflect tissue concentrations of the Klotho protein, which cannot be obtained for analysis in the absence of a clinical indication for muscle biopsy. Additionally, based on the inclusion criteria of the present study, the impact of S-Klotho on mortality in diseased populations cannot be determined; the data only allow conclusions to be drawn regarding its association with cardiometabolic risk factors and insulin sensitivity in healthy people.

In conclusion, the present work shows plasma S-Klotho to be inversely associated with cardiometabolic risk, and positively related to insulin sensitivity, in healthy sedentary middle-aged adults, independent of their actual age, cardiorespiratory fitness, physical activity levels, or dietary intake; no such association was observed for healthy young, sedentary adults. Further studies are needed to examine whether plasma S-Klotho can be used as a predictor of cardiometabolic disease at certain times of life, and whether changes in cardiovascular risk in response to different interventions (e.g., physical exercise or nutritional strategies) are mediated via changes in S-Klotho.

## MATERIALS AND METHODS

### Study design and participants

The study subjects of this cross-sectional study were 214 healthy, sedentary adults (~64% women) (Supplementary Figures 1 and 2). Data were collected during the baseline assessments of two randomized controlled trials: the FIT-AGEING study (https://clinicaltrials.gov ID: NCT03334357; n=74 [~50% women]) [35], and the ACTIBATE study (https://clinicaltrials.gov ID: NCT02365129; n=140 [~70% women]) [36]. Subjects were recruited via social networks, electronic media, and leaflets. Details regarding inclusion and exclusion criteria are described elsewhere [35–37]. Briefly, the subjects of the FIT-AGEING study were healthy 40-65 year-olds (middle-aged adults) and those of the ACTIBATE study 18-25 year-olds in (young adults), all of whom were physically inactive (<20 min of moderate-vigorous physical activity on <3 days/week) and had a stable body weight (<3 kg changes during the 12 weeks prior to assessment). The study protocol and methodology were designed according to the last revised version of the Declaration of Helsinki (2013). The Human Research Ethics Committee of the *Junta de Andalucía* [0838-N-2017] and the Human Research Ethics Committee of the University of Granada (nº 924) approved the respective studies, and all participants provided written informed consent to be included.

### Procedures

### Anthropometry

Body mass index was calculated from weight (kg) and height (m^2^); the required measurements were taken using a model 799 stadiometer (Seca, Hamburg, Germany). Waist circumference was assessed at the mid-point between the bottom of the rib cage and the iliac crest at the end of normal expiration (mean of three measurements), following the standard procedures of the International Society for the Advancement of Kinanthropometry (ISAK) [38].

### Blood pressure

Blood pressure was recorded with subjects resting on the right arm in a sitting position, using an HEM 705 CP automatic monitor (Omron Healthcare Co., Kyoto, Japan) following the guidelines of the European Heart Society [39]. Readings were taken twice and the mean recorded. Mean blood pressure was defined as systolic blood pressure minus 1/3 of the diastolic blood pressure [39].

### Blood samples

Blood for analysis was obtained from the antecubital vein after an overnight fast and a minimum 10 min rest in a supine position. Samples were collected in prechilled ethylene diamine tetra-acetic acid-containing tubes (Vacutainer SST, Becton Dickinson, Plymouth, UK) and immediately centrifuged (i.e. 15 minutes at 3,000 rpm), aliquoted and stored at −80°C until analysis. S-Klotho was determined using a solid-phase sandwich enzyme-linked immunosorbent assay kit (Demeditec, Kiel, Germany), following the manufacturer's instructions. To determine the intra- and inter-assay coefficients of variation, two different doses of purified S-Klotho were measured; values ranged from 3% to 10%. Glucose and insulin were respectively assessed using a model AU5800 spectrophotometer (Beckman Coulter, Brea, CA, USA) and by chemiluminescence immunoassay involving UniCel DxI 800 paramagnetic particles (Beckman Coulter, Brea, CA, USA). Total cholesterol, HDL-C, and triglycerides were measured using the same spectrophotometric apparatus, and LDL-C calculated as (total cholesterol) – (HDL-C) – 0.45 * (triglycerides). ALT and γ-GT were determined using an absorption spectrophotometer (Beckman Coulter, Brea, CA, USA). The insulin/glucose, LDL-C/HDL-C ratio, and the triglycerides/HDL-C ratios were also calculated. To note is that the methodology for collection and processing blood samples in the FIT-AGEING and ACTIBATE studies involved the same researchers and technicians and followed the same procedures.

### Cardiometabolic risk indices

Sex-specific cardiometabolic risk scores were calculated based on the clinical variables suggested by the International Diabetes Federation and the Adult Treatment Panel III for defining metabolic syndrome, i.e., waist circumference, mean blood pressure, plasma glucose, HDL-C and triglycerides [40], represented in a standardized fashion as (value-mean)/standard deviation. To indicate greater risk via increasing values, the standardized HDL-C values were multiplied by -1. Cardiometabolic risk scores were calculated as the sum of these 5 standardized values divided by 5, obtaining a mean of 0 and a standard deviation of 1 by definition, understanding lower values to represent a better cardiometabolic risk profile. This score was independently calculated for both original trial population and sex.

Insulin sensitivity was estimated via the quantitative insulin sensitivity check index (QUICKI) [41] and the homeostatic model assessment of insulin resistance index (HOMA) [42]:

$$QUICKI=\frac{1}{Loge\;(Insulin)+Loge(Glucose)}$$

and

$$HOMA=\frac{Insulin\;*\;Glucose}{22.5}$$

The fatty liver index, a surrogate marker of fatty liver function in non-alcoholic individuals, was calculated using a previously validated equation [43]:

$$\begin{array}{l} Fatty\;liver\;index=(e^{0.953*loge(triglycerides)+0.139*body\;mass\;index}\\ \;\;\;\;\;\;\;\;{\;}^{+0.718*loge(\gamma -GT)+0.053*waist\;circunference-15.745)})\\ \;\;\;\;\;\;\;\;\;*100 \end{array}$$

### Dietary intake

Dietary intake was assessed via three non-consecutive 24 h recalls. Energy (kcal/day), fat, carbohydrate and protein intakes (% energy intake) [44] were determined using EvalFINUT® software.

### Sedentary behaviour and physical activity level

Sedentary behaviour and physical activity were objectively assessed via triaxial accelerometry (ActiGraph GT3X+, Pensacola, FL, US) using an accelerometer worn on each subject's non-dominant wrist for 24 h/day over 7 consecutive days. Data were exported using ActiLife v.6.13.3 software (ActiGraph, Pensacola, FL, US), and processed using the GGIR v.1.6-0 package (https://cran.r-project.org/web/packages/GGIR/index.html) in R v.3.1.2 (https://cran.r-project.org/bin/windows/base/old/3.1.2/) [45]. Sedentary time and moderate-vigorous physical activity levels were then computed.

### Cardiorespiratory fitness

Maximum oxygen uptake (VO_2_max) was determined by indirect calorimetry using a maximum treadmill graded exercise test extensively described elsewhere [37, 46]. Briefly, subjects walked at 5.3 km/h, increasing the slope by 1% every minute until self-reported exhaustion. Subjects were instructed to fast for 3 h prior to the test, not to consume any drugs during the previous 48 h, and not to perform any moderate or vigorous physical activity for 24 h and 48 h before the test respectively. VO_2_max criteria was deemed reached when (i) a respiratory exchange ratio of ≥1.1 was attained, (ii) a plateau in VO_2_ was observed (change of <100 ml/min in the last 30 s), and (iii) a heart rate within 10 bpm of the age-predicted maximum was reached. Peak oxygen uptake was recorded during the exercise test if one or more than these three criteria were not met [47].

### Statistical analysis

The normal distribution of all variables was confirmed using the Shapiro-Wilk test, Q-Q plots, and visual checking of histograms. Descriptive variables are reported as means±SD. Unpaired Student t- tests were used to analyze differences between men and women. Given that the interaction *sex x original trial population* (i.e., FIT-AGEING or ACTIBATE) had a significant influence on many measured outcomes (P<0.05), the results for men and women in both original trials were analyzed separately.

A simple linear regression model (Model 0) was used to study the association between S-Klotho and cardiometabolic risk score, and the QUICKI and HOMA results. Multiple linear regression models were used to test these associations, adjusting for age (Model 1), energy intake (Model 2) and VO_2_max (Model 3). Similar analyses were conducted to study the relationship between S-Klotho and cardiometabolic risk factors. Calculations were made using the Statistical Package for the Social Sciences v.22.0, (IBM SPSS Statistics, IBM Corporation), and plots drawn using GraphPad Prism 5 software (GraphPad Software, San Diego, CA, USA). Significance was set at P<0.05.

## Supplementary Material

Supplementary Figures

Supplementary Table 1

Supplementary Table 2
