# Supplementary material for: Relationship between plasma S-Klotho and cardiometabolic risk in sedentary adults
Source: Aging (Albany NY). 2020 Jan 20;12(3):2698–710. doi: 10.18632/aging.102771 (PMC7041759; doi:10.18632/aging.102771)
Supplement: Supplementary Figures [file aging-12-102771-s003..pdf]

SUPPLEMENTARY FIGURES

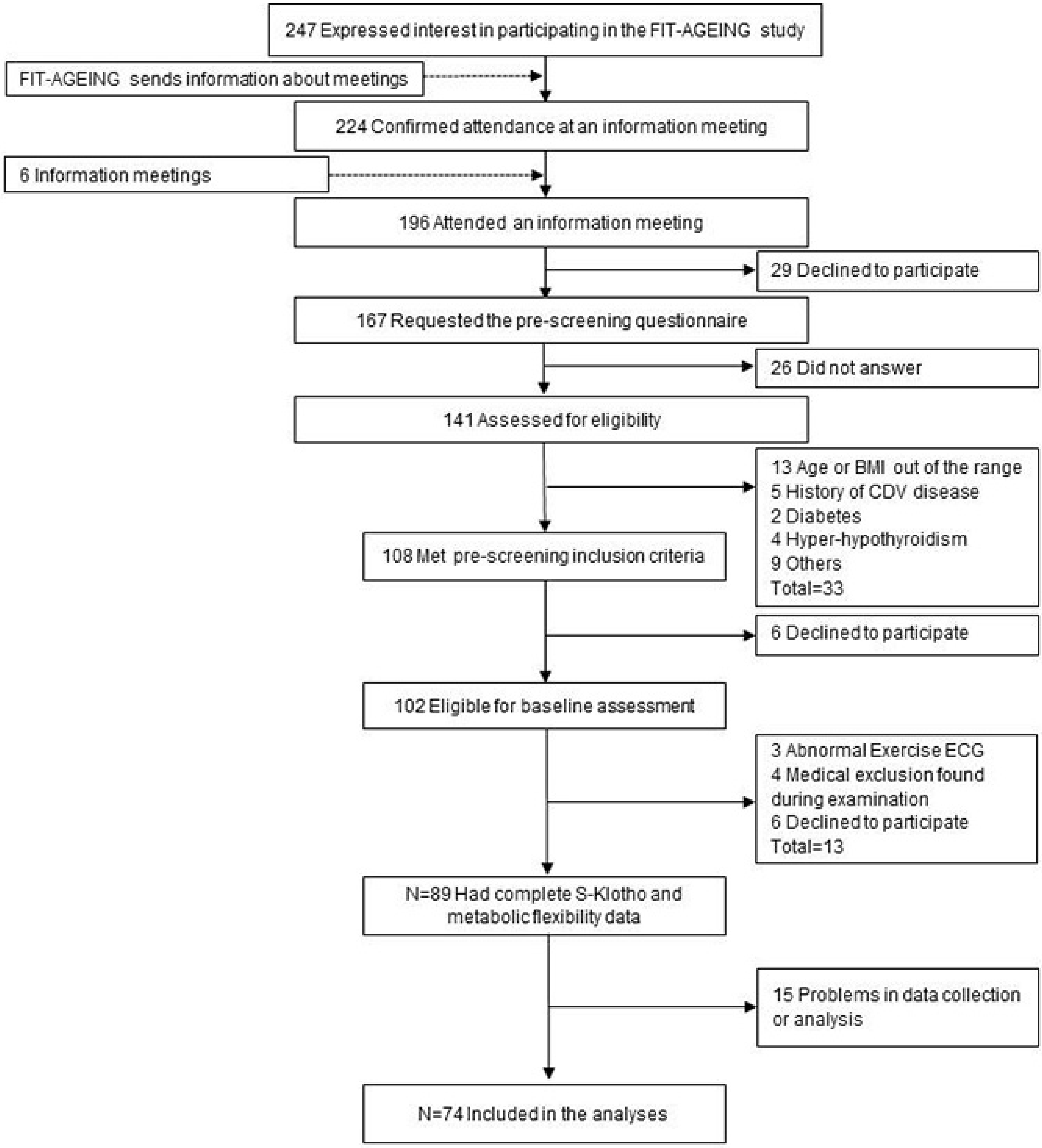

Supplementary Figure 1. Flow-chart of the FIT-AGEING study as followed for the present work. BMI: body mass index, CVD: cardiovascular disease, ECG: electrocardiogram.

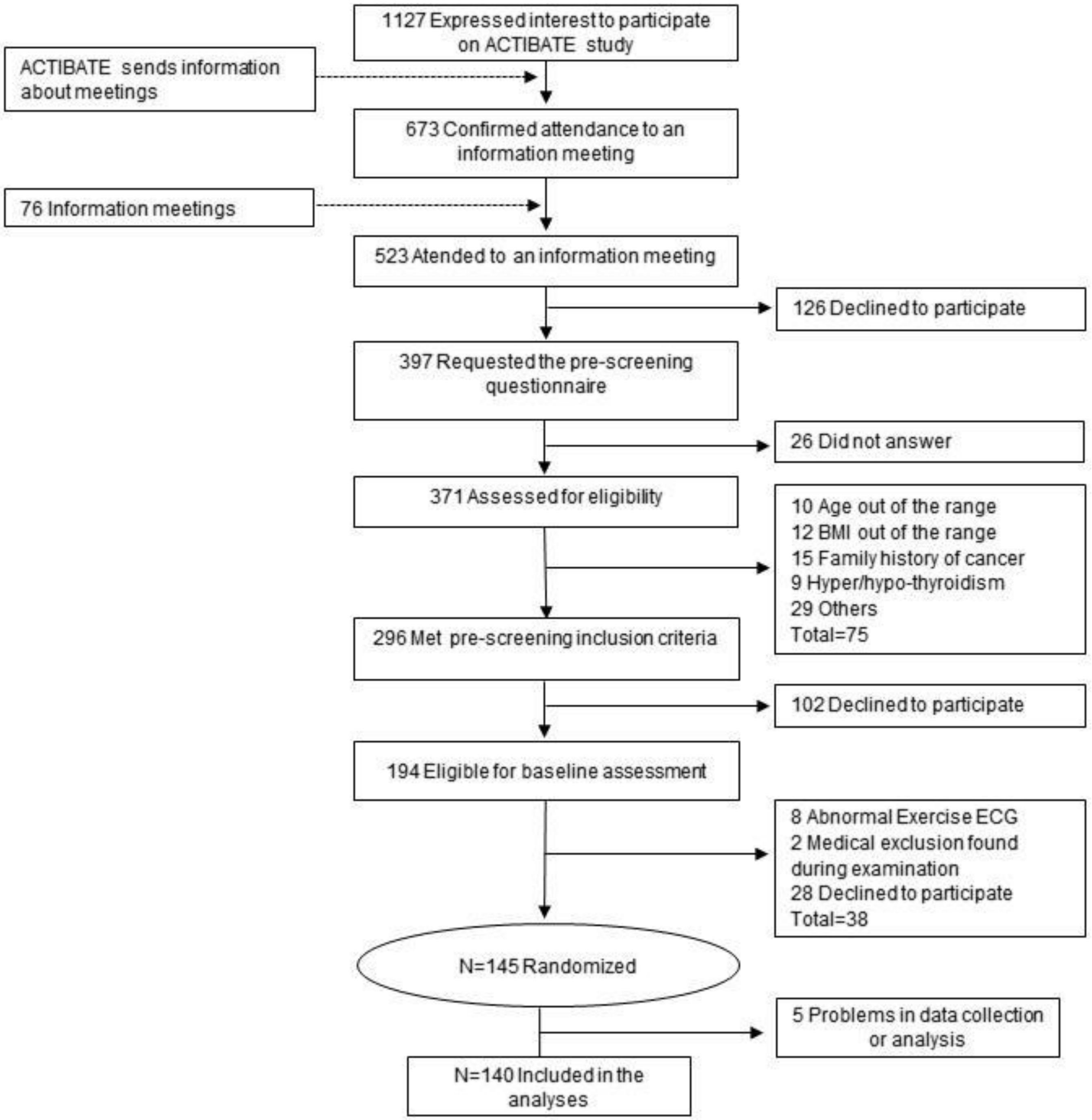

Supplementary Figure 2. Flow-chart of the ACTIBATE study as followed for the present work. BMI: body mass index, CVD: cardiovascular disease, ECG: electrocardiogram.
