# Supplementary material for: Relationship between plasma S-Klotho and cardiometabolic risk in sedentary adults
Source: Aging (Albany NY). 2020 Jan 20;12(3):2698–710. doi: 10.18632/aging.102771 (PMC7041759; doi:10.18632/aging.102771)
Supplement: Supplementary Table 1 [file aging-12-102771-s001..docx]

| Supplementary Table 1. Descriptive characteristics of the study subjects. | | | | | | | | | | | | | | | | | | |
| --- | --- | --- | --- | --- | --- | --- | --- | --- | --- | --- | --- | --- | --- | --- | --- | --- | --- | --- |
|  | **Middle-aged adults** | | | | | | | | | **Young adults** | | | | | | | | |
|  | **N** | **All** | | **N** | **Men** | | **N** | **Women** | | **N** | **All** | | **N** | **Men** | | **N** | **Women** | |
| Age (years) | 74 | 53.7 | (5.1) | 35 | 54.4 | (5.3) | 39 | 53.0 | (5.0) | 140 | 22.1 | (2.2) | 42 | 22.5 | (2.2) | 98 | 22.0 | (2.2) |
| S-Klotho (pg/ml) | 73 | 775.3 | (363.7) | 34 | 814.1 | (452.2) | 39 | 741.4 | (265.6) | 140 | 823.1 | (556.9) | 42 | 637.3 | (434.7) | 98 | 902.7 | (585.9)* |
| **Anthropometry** | | | | | | | | | | | | | | | | | | |
| Weight (kg) | 74 | 75.7 | (15.0) | 35 | 87.4 | (11.0) | 39 | 65.3 | (9.3)* | 140 | 70.8 | (16.8) | 42 | 85.1 | (18.0) | 98 | 64.7 | (11.8)* |
| Height (cm) | 74 | 167.8 | (9.8) | 35 | 175.8 | (6.5) | 39 | 160.7 | (6.1)* | 140 | 167.8 | (8.5) | 42 | 176.0 | (6.5) | 98 | 164.3 | (6.6)* |
| Body mass index (kg/m^2^) | 74 | 26.7 | (3.8) | 35 | 28.3 | (3.6) | 39 | 25.3 | (3.3)* | 140 | 25.0 | (4.7) | 42 | 27.4 | (5.5) | 98 | 23.9 | (3.9)* |
| Waist circumference (cm) | 74 | 95.1 | (11.7) | 35 | 102.7 | (8.8) | 39 | 88.2 | (9.7)* | 137 | 80.9 | (14.2) | 42 | 91.2 | (15.8) | 95 | 76.4 | (10.7)* |
| **Blood pressure** | | | | | | | | | | | | | | | | | | |
| Systolic blood pressure (mm Hg) | 70 | 127.1 | (15.8) | 32 | 134.3 | (13.8) | 38 | 120.9 | (14.8)* | 138 | 116.5 | (12.0) | 41 | 126.5 | (11.7) | 97 | 112.2 | (9.4)* |
| Diastolic blood pressure (mm Hg) | 70 | 81.1 | (11.7) | 32 | 85.2 | (10.9) | 38 | 77.6 | (11.4)* | 138 | 71.1 | (7.2) | 41 | 73.3 | (8.4) | 97 | 70.2 | (6.4)* |
| Mean blood pressure (mm Hg) | 70 | 104.1 | (13.1) | 32 | 109.7 | (11.7) | 38 | 99.3 | (12.5)* | 138 | 93.8 | (8.6) | 41 | 99.9 | (9.0) | 97 | 91.2 | (7.1)* |
| **Glycaemic metabolism** | | | | | | | | | | | | | | | | | | |
| Glucose (mg/dL) | 73 | 93.5 | (11.2) | 34 | 94.8 | (13.4) | 38 | 92.4 | (8.8) | 138 | 87.7 | (6.8) | 42 | 89.6 | (8.3) | 96 | 86.9 | (5.9)* |
| Insulin (UI/mL) | 73 | 8.2 | (5.6) | 34 | 8.8 | (6.7) | 38 | 7.6 | (4.6) | 138 | 8.6 | (5.8) | 42 | 10.2 | (8.6) | 96 | 8.0 | (4.0)* |
| Insulin glucose ratio | 73 | 12.7 | (7.5) | 34 | 13.2 | (8.1) | 38 | 12.3 | (7.1) | 138 | 14.5 | (7.7) | 42 | 16.2 | (10.6) | 96 | 13.7 | (6.0) |
| QUICKI | 73 | 0.362 | (0.036) | 34 | 0.357 | (0.039) | 38 | 0.365 | (0.033) | 138 | 0.156 | (0.013) | 42 | 0.154 | (0.015) | 96 | 0.157 | (0.012) |
| HOMA | 73 | 1.95 | (1.65) | 34 | 2.17 | (2.09) | 38 | 1.76 | (1.14) | 138 | 1.92 | (1.56) | 42 | 2.38 | (2.35) | 96 | 1.74 | (0.98)* |
| **Lipid metabolism** | | | | | | | | | | | | | | | | | | |
| Total cholesterol (mg/dL) | 73 | 207.5 | (33.5) | 34 | 203.8 | (36.7) | 39 | 210.6 | (30.5) | 138 | 164.4 | (32.1) | 42 | 162.3 | (37.6) | 96 | 165.4 | (29.5) |
| Triglycerides (mg/dL) | 73 | 136 | (67.8) | 34 | 147.4 | (83.8) | 39 | 126.1 | (49.1) | 138 | 83.9 | (51.2) | 42 | 95.1 | (61.0) | 96 | 78.9 | (45.9) |
| HDL-C (mg/dL) | 73 | 59.1 | (12.8) | 34 | 55.5 | (12.7) | 39 | 62.3 | (12.2)* | 138 | 52.6 | (11.2) | 42 | 45.7 | (8.2) | 96 | 55.6 | (11.0)* |
| LDL-C (mg/dL) | 73 | 126.6 | (29.3) | 34 | 127.8 | (31.9) | 39 | 125.5 | (27.2) | 138 | 95.7 | (26.2) | 42 | 98.0 | (30.2) | 96 | 94.8 | (24.4) |
| LDL-C/HDL-C | 73 | 2.30 | (0.91) | 34 | 2.48 | (0.96) | 39 | 2.10 | (0.80) | 138 | 0.59 | (0.22) | 42 | 0.52 | (0.27) | 96 | 0.62 | (0.19)* |
| Triglycerides/HDL-C | 73 | 2.58 | (1.89) | 34 | 3.05 | (2.36) | 39 | 2.17 | (1.25)* | 138 | 1.71 | (1.29) | 42 | 2.24 | (1.70) | 96 | 1.47 | (0.99)* |
| **Cardiometabolic risk score** | 70 | 0.002 | (0.34) | 32 | 0.026 | (0.384) | 38 | -0.017 | (0.302)* | 134 | -0.002 | (0.400) | 41 | 0.001 | (0.458) | 93 | -0.003 | (0.374) |
| **Liver function** | | | | | | | | | | | | | | | | | | |
| ALT (IU/L) | 73 | 23.3 | (12.5) | 34 | 29.0 | (13.6) | 39 | 18.3 | (9.0)* | 137 | 19.5 | (19.7) | 42 | 30.2 | (31.4) | 95 | 14.7 | (7.5)* |
| γ-GT (IU/L) | 73 | 34.4 | (23.2) | 34 | 41.0 | (23.3) | 39 | 28.7 | (21.9)* | 137 | 19.7 | (21.6) | 42 | 31.1 | (34.7) | 95 | 14.7 | (8.1)* |
| Fatty liver index | 73 | 50.0 | (26.0) | 34 | 66.7 | (20.2) | 39 | 35.5 | (21.6) | 135 | 20.9 | (25.8) | 42 | 38.9 | (33.2) | 93 | 12.8 | (16.1)* |
| **Dietary intake** | | | | | | | | | | | | | | | | | | |
| Energy (kcal/day) | 73 | 2134 | (688) | 34 | 2374 | (838) | 39 | 1913 | (414)* | 140 | 1902 | (508) | 42 | 2096 | (551) | 98 | 1819 | (466)* |
| Fat (g/day) | 73 | 38.8 | (10.1) | 34 | 38.0 | (5.9) | 39 | 39.5 | (12.8) | 140 | 84.7 | (27.3) | 42 | 91.3 | (30.2) | 98 | 81.9 | (25.6) |
| Carbohydrate (g/day) | 73 | 52.4 | (29.3) | 34 | 49.0 | (15.6) | 39 | 55.6 | (37.8) | 140 | 203.2 | (66.1) | 42 | 218.2 | (74.0) | 98 | 196.8 | (61.7) |
| Protein (g/day) | 73 | 22.5 | (21.0) | 34 | 19.1 | (8.8) | 39 | 25.7 | (27.7) | 140 | 76.0 | (21.0) | 42 | 90.3 | (22.5) | 98 | 69.9 | (17.1)* |
| Ethanol (g/day) | 73 | 11.2 | (13.1) | 34 | 16.2 | (16.0) | 39 | 6.6 | (7.2)* | 140 | 13.2 | (17.2) | 42 | 18.9 | (18.2) | 98 | 9.8 | (9.1)* |
| **Sedentary behaviour and physical activity levels** | | | | | | | | | | | | | | | | | | |
| Sedentary time (min/day) | 71 | 745.9 | (84.2) | 34 | 770.0 | (80.3) | 37 | 723.7 | (82.6)* | 139 | 934.1 | (51.2) | 42 | 946.9 | (58.5) | 97 | 928.6 | (46.9) |
| MVPA (min/day) | 71 | 96.1 | (35.4) | 34 | 96.6 | (35.5) | 37 | 95.5 | (35.8) | 139 | 60.2 | (23.9) | 42 | 56.8 | (22.7) | 97 | 61.7 | (24.4) |
| **Cardiorespiratory fitness** | | | | | | | | | | | | | | | | | | |
| VO_2_max (ml/min) | 71 | 2339.2 | (657.2) | 34 | 2915.4 | (373.2) | 37 | 1809.7 | (332.5)* | 135 | 2887.2 | (756.3) | 40 | 3716.5 | (690.6) | 95 | 2538.0 | (449.0)* |
| VO_2_max (ml/kg/min) | 71 | 30.5 | (5.6) | 34 | 33.3 | (4.5) | 37 | 27.9 | (5.3)* | 135 | 41.1 | (8.1) | 40 | 44.8 | (9.4) | 95 | 39.5 | (7.0)* |

Data are means (standard deviation). *Significant differences between sexes (Student unpaired t test; P<0.05). Abbreviations: S-Klotho; shed form of the Klotho protein, QUICKI quantitative insulin sensitivity check index, HOMA homeostasis model assessment index, HDL-C high-density lipoprotein cholesterol, LDL-C low-density lipoprotein cholesterol, ALT alanine transaminase, γ-GT; γ-glutamyl transferase, MVPA; Moderate-vigorous intensity physical activity levels, VO_2_max; Maximum oxygen uptake.
