# Supplementary material for: Relationship between plasma S-Klotho and cardiometabolic risk in sedentary adults
Source: Aging (Albany NY). 2020 Jan 20;12(3):2698–710. doi: 10.18632/aging.102771 (PMC7041759; doi:10.18632/aging.102771)
Supplement: Supplementary Table 2 [file aging-12-102771-s002..docx]

| **Supplementary Table 2.** Association between S-Klotho and cardiometabolic risk variables (Model 0, unadjusted), adjusted for age (Model 1), energy intake (Model 2) and cardiorespiratory fitness (Model 3). | | | | | | | | | | | | | | | | | | | |
| --- | --- | --- | --- | --- | --- | --- | --- | --- | --- | --- | --- | --- | --- | --- | --- | --- | --- | --- | --- |
|  | **Middle-aged adults Young adults** | | | | | | | | | | | | | | | | | | |
|  | Men | | | | | | Women | | | | | | Men | | | | Women | | |
|  | β | | R^2^ | | P | | β | | R^2^ | | P | | β | | R^2^ | P | β | R^2^ | P |
| Weight (kg) |  | |  | |  | |  | |  | |  | |  | |  |  |  |  |  |
| Model 0 | 0.294 | | 0.087 | | 0.091 | | 0.210 | | 0.044 | | 0.200 | | -0.171 | | 0.029 | 0.280 | 0.062 | 0.004 | 0.547 |
| Model 1 | 0.116 | | 0.105 | | 0.684 | | 0.043 | | 0.081 | | 0.838 | | -0.186 | | 0.141 | 0.217 | 0.065 | 0.004 | 0.543 |
| Model 2 | 0.300 | | 0.090 | | 0.091 | | 0.208 | | 0.044 | | 0.262 | | -0.167 | | 0.103 | 0.276 | 0.059 | 0.020 | 0.560 |
| Model 3 | **0.491** | | **0.439** | | **0.002** | | **0.402** | | **0.273** | | **0.015** | | -0.011 | | 0.396 | 0.935 | 0.126 | 0.216 | 0.178 |
| Waist circumference (cm) |  | |  | |  | |  | |  | |  | |  | |  |  |  |  |  |
| Model 0 | 0.334 | | 0.112 | | 0.054 | | 0.079 | | 0.006 | | 0.634 | | -0.167 | | 0.028 | 0.292 | 0.036 | 0.001 | 0.727 |
| Model 1 | 0.197 | | 0.112 | | 0.487 | | -0.047 | | 0.028 | | 0.828 | | -0.184 | | 0.163 | 0.218 | 0.060 | 0.005 | 0.611 |
| Model 2 | 0.344 | | 0.122 | | 0.051 | | 0.218 | | 0.102 | | 0.225 | | .0.164 | | 0.070 | 0.294 | 0.034 | 0.003 | 0.742 |
| Model 3 | **0.558** | | **0.512** | | **<0.001** | | 0.306 | | 0.257 | | 0.063 | | 0.006 | | 0.434 | 0.962 | 0.096 | 0.208 | 0.315 |
| Systolic blood pressure (mm Hg) |  | |  | |  | |  | |  | |  | |  | |  |  |  |  |  |
| Model 0 | **-0.767** | | **0.588** | | **<0.001** | | **-0.487** | | **0.237** | | **0.003** | | -0.201 | | 0.041 | 0.207 | 0.027 | 0.001 | 0.794 |
| Model 1 | **-0.868** | | **0.594** | | **<0.001** | | -0.108 | | 0.388 | | 0.541 | | -0.201 | | 0.041 | 0.213 | 0.007 | 0.005 | 0.951 |
| Model 2 | **-0.775** | | **0.591** | | **<0.001** | | **-0.634** | | **0.336** | | **<0.001** | | -0.203 | | 0.046 | 0.207 | 0.026 | 0.003 | 0.799 |
| Model 3 | **-0.708** | | **0.603** | | **<0.001** | | **-0.505** | | **0.240** | | **0.004** | | -0.178 | | 0.141 | 0.272 | 0.065 | 0.077 | 0.526 |
| Diastolic blood pressure (mm Hg) |  | |  | |  | |  | |  | |  | |  | |  |  |  |  |  |
| Model 0 | **-0.618** | | **0.381** | | **<0.001** | | **-0.359** | | **0.129** | | **0.031** | | -0.095 | | 0.009 | 0.554 | -0.037 | 0.001 | 0.720 |
| Model 1 | **-0.565** | | **0.383** | | **<0.001** | | -0.005 | | 0.268 | | 0.982 | | -0.106 | | 0.125 | 0.490 | -0.043 | 0.002 | 0.688 |
| Model 2 | **-0.613** | | **0.382** | | **<0.001** | | **-0.529** | | **0.247** | | **0.004** | | -0.095 | | 0.009 | 0.559 | -0.037 | 0.002 | 0.723 |
| Model 3 | **-0.511** | | **0.431** | | **0.004** | | **-0.390** | | **0.137** | | **0.030** | | -0.073 | | 0.116 | 0.655 | -0.002 | 0.066 | 0.986 |
| Mean blood pressure (mm Hg) |  | |  | |  | |  | |  | |  | |  | |  |  |  |  |  |
| Model 0 | **-0.740** | | **0.547** | | **<0.001** | | **-0.453** | | **0.205** | | **0.006** | | -0.176 | | 0.031 | 0.272 | 0.001 | 0.001 | 0.991 |
| Model 1 | **-0.775** | | **0.548** | | **0.001** | | -0.062 | | 0.365 | | 0.752 | | -0.180 | | 0.055 | 0.260 | -0.015 | 0.003 | 0.887 |
| Model 2 | **-0.742** | | **0.547** | | **<0.001** | | **-0.616** | | **0.232** | | **0.001** | | -0.177 | | 0.033 | 0.275 | 0.001 | 0.001 | 0.984 |
| Model 3 | **-0.655** | | **0.578** | | **<0.001** | | **-0.478** | | **0.210** | | **0.006** | | -0.148 | | 0.151 | 0.356 | 0.042 | 0.090 | 0.677 |
| Glucose (mg/dL) |  | |  | |  | |  | |  | |  | |  | |  |  |  |  |  |
| Model 0 | -0.056 | | 0.003 | | 0.755 | | 0.011 | | 0.000 | | 0.948 | | -0.236 | | 0.055 | 0.133 | -0.012 | 0.001 | 0.905 |
| Model 1 | -0.043 | | 0.003 | | 0.886 | | -0.084 | | 0.012 | | 0.701 | | -0.240 | | 0.063 | 0.131 | -0.010 | 0.001 | 0.923 |
| Model 2 | -0.025 | | 0.094 | | 0.883 | | 0.007 | | 0.004 | | 0.971 | | -0.235 | | 0.059 | 0.139 | -0.017 | 0.028 | 0.868 |
| Model 3 | -0.024 | | 0.020 | | 0.902 | | 0.042 | | 0.002 | | 0.822 | | -0.140 | | 0.226 | 0.353 | 0.012 | 0.046 | 0.911 |
| Insulin (UI/mL) |  | |  | |  | |  | |  | |  | |  | |  |  |  |  |  |
| Model 0 | **-0.423** | | **0.179** | | **0.013** | | **-0.504** | | **0.254** | | **0.001** | | -0.153 | | 0.024 | 0.332 | 0.075 | 0.006 | 0.466 |
| Model 1 | -0.330 | | 0.184 | | 0.229 | | **-0.585** | | **0.263** | | **0.004** | | -0.161 | | 0.051 | 0.309 | 0.051 | 0.013 | 0.636 |
| Model 2 | **-0.396** | | **0.251** | | **0.016** | | **-0.480** | | **0.250** | | **0.005** | | -0.153 | | 0.026 | 0.340 | 0.072 | 0.018 | 0.484 |
| Model 3 | **-0.391** | | **0.231** | | **0.031** | | **-0.546** | | **0.257** | | **0.002** | | -0.010 | | 0.398 | 0.939 | 0.113 | 0.170 | 0.248 |
| Insulin glucose ratio |  | |  | |  | |  | |  | |  | |  | |  |  |  |  |  |
| Model 0 | **-0.492** | | **0.242** | | **0.003** | | **-0.534** | | **0.285** | | **<0.001** | | -0.139 | | 0.019 | 0.381 | 0.100 | 0.010 | 0.334 |
| Model 1 | -0.374 | | 0.250 | | 0.157 | | **-0.593** | | **0.290** | | **0.003** | | -0.146 | | 0.044 | 0.358 | 0.075 | 0.017 | 0.483 |
| Model 2 | **-0.472** | | **0.236** | | **0.004** | | **-0.508** | | **0.242** | | **0.003** | | -0.138 | | 0.024 | 0.389 | 0.098 | 0.015 | 0.346 |
| Model 3 | **-0.444** | | **0.319** | | **0.010** | | **-0.565** | | **0.276** | | **0.001** | | 0.002 | | 0.408 | 0.988 | 0.135 | 0.167 | 0.168 |
| Total cholesterol (mg/dL) |  | |  | |  | |  | |  | |  | |  | |  |  |  |  |  |
| Model 0 | **-0.704** | | **0.496** | | **<0.001** | | **-0.516** | | **0.266** | | **0.001** | | -0.149 | | 0.022 | 0.348 | -0.101 | 0.010 | 0.330 |
| Model 1 | **-0.497** | | **0.520** | | **0.022** | | **-0.641** | | **0.287** | | **0.001** | | -0.155 | | 0.040 | 0.330 | -0.052 | 0.038 | 0.627 |
| Model 2 | **-0.701** | | **0.496** | | **<0.001** | | **-0.515** | | **0.282** | | **0.002** | | -0.148 | | 0.024 | 0.355 | -0.140 | 0.029 | 0.310 |
| Model 3 | **-0.726** | | **0.523** | | **<0.001** | | **-0.550** | | **0.355** | | **0.001** | | -0.037 | | 0.187 | 0.811 | -0.129 | 0.028 | 0.223 |
| **Table 2.** Continued. | | | | | | | | | | | | | | | | | | | |
|  | | **Middle-aged adults Young adults** | | | | | | | | | | | | | | | | | |
|  | | Men | | | | | | Women | | | | | | Men | | | Women | | |
|  | | β | | R^2^ | | P | | β | | R^2^ | | P | | β | R^2^ | P | β | R^2^ | P |
| Triglycerides (mg/dL) | |  | |  | |  | |  | |  | |  | |  |  |  |  |  |  |
| Model 0 | | **-0.634** | | **0.401** | | **<0.001** | | **-0.428** | | **0.183** | | **0.007** | | -0.114 | 0.013 | 0.472 | -0.054 | 0.003 | 0.601 |
| Model 1 | | **-0.748** | | **0.409** | | **<0.001** | | -0.355 | | 0.190 | | 0.081 | | -0.122 | 0.045 | 0.439 | -0.021 | 0.016 | 0.848 |
| Model 2 | | **-0.613** | | **0.445** | | **<0.001** | | -0.302 | | 0.244 | | 0.071 | | -0.115 | 0.015 | 0.475 | -0.056 | 0.008 | 0.589 |
| Model 3 | | **-0.598** | | **0.390** | | **0.001** | | **-0.374** | | **0.194** | | **0.031** | | 0.006 | 0.277 | 0.965 | -0.040 | 0.048 | 0.698 |
| HDL-C (mg/dL) | |  | |  | |  | |  | |  | |  | |  |  |  |  |  |  |
| Model 0 | | **0.852** | | **0.726** | | **<0.001** | | **0.378** | | **0.372** | | **0.020** | | 0.164 | 0.027 | 0.301 | 0.008 | 0.001 | 0.941 |
| Model 1 | | **0.997** | | **0.738** | | **<0.001** | | 0.266 | | 0.153 | | 0.196 | | 0.169 | 0.041 | 0.288 | 0.021 | 0.002 | 0.847 |
| Model 2 | | **0.862** | | **0.737** | | **<0.001** | | 0.285 | | 0.160 | | 0.104 | | 0.162 | 0.037 | 0.308 | 0.007 | 0.001 | 0.949 |
| Model 3 | | **0.917** | | **0.790** | | **<0.001** | | **0.589** | | **0.307** | | **0.001** | | 0.147 | 0.043 | 0.382 | -0.033 | 0.113 | 0.739 |
| LDL-C (mg/dL) | |  | |  | |  | |  | |  | |  | |  |  |  |  |  |  |
| Model 0 | | **-0.718** | | **0.516** | | **<0.001** | | **-0.329** | | **0.108** | | **0.041** | | -0.188 | 0.035 | 0.234 | -0.106 | 0.011 | 0.303 |
| Model 1 | | **-0.396** | | **0.575** | | **0.050** | | -0.215 | | 0.125 | | 0.302 | | -0.194 | 0.056 | 0.220 | -0.066 | 0.030 | 0.536 |
| Model 2 | | **-0.713** | | **0.519** | | **<0.001** | | -0.267 | | 0.122 | | 0.098 | | -0.187 | 0.043 | 0.241 | -0.110 | 0.029 | 0.286 |
| Model 3 | | **-0.757** | | **0.551** | | **<0.001** | | **-0.415** | | **0.239** | | **0.015** | | -0.086 | 0.165 | 0.583 | -0.122 | 0.015 | 0.251 |
| LDL-C/HDL-C | |  | |  | |  | |  | |  | |  | |  |  |  |  |  |  |
| Model 0 | | **-0.858** | | **0.736** | | **<0.001** | | **-0.444** | | **0.197** | | **0.005** | | 0.066 | 0.004 | 0.679 | 0.061 | 0.004 | 0.554 |
| Model 1 | | **-0.736** | | **0.744** | | **<0.001** | | -0.366 | | 0.205 | | 0.070 | | 0.074 | 0.037 | 0.639 | 0.057 | 0.004 | 0.596 |
| Model 2 | | **-0.854** | | **0.737** | | **<0.001** | | **-0.379** | | **0.212** | | **0.028** | | 0.065 | 0.013 | 0.686 | 0.062 | 0.006 | 0.547 |
| Model 3 | | **-0.814** | | **0.730** | | **<0.001** | | **-0.536** | | **0.131** | | **0.001** | | -0.022 | 0.130 | 0.892 | 0.047 | 0.032 | 0.653 |
| Triglycerides/HDL-C | |  | |  | |  | |  | |  | |  | |  |  |  |  |  |  |
| Model 0 | | **-0.654** | | **0.428** | | **<0.001** | | **-0.480** | | **0.230** | | **0.002** | | -0.120 | 0.014 | 0.451 | -0.043 | 0.002 | 0.678 |
| Model 1 | | **-0.760** | | **0.434** | | **0.002** | | -0.374 | | 0.245 | | 0.058 | | -0.129 | 0.057 | 0.411 | -0.016 | 0.011 | 0.884 |
| Model 2 | | **-0.637** | | **0.456** | | **<0.001** | | **-0.357** | | **0.287** | | **0.029** | | -0.120 | 0.014 | 0.457 | -0.045 | 0.007 | 0.665 |
| Model 3 | | **-0.650** | | **0.430** | | **<0.001** | | **-0.481** | | **0.268** | | **0.005** | | -0.013 | 0.211 | 0.933 | -0.016 | 0.093 | 0.873 |
| ALT (IU/L) | |  | |  | |  | |  | |  | |  | |  |  |  |  |  |  |
| Model 0 | | 0.186 | | 0.035 | | 0.292 | | -0.209 | | 0.044 | | 0.201 | | -0.206 | 0.042 | 0.192 | -0.113 | 0.013 | 0.275 |
| Model 1 | | 0.237 | | 0.036 | | 0.424 | | -0.473 | | 0.138 | | 0.226 | | -0.215 | 0.086 | 0.168 | -0.164 | 0.044 | 0.125 |
| Model 2 | | 0.182 | | 0.036 | | 0.313 | | -0.161 | | 0.049 | | 0.383 | | -0.203 | 0.087 | 0.192 | -0.111 | 0.020 | 0.287 |
| Model 3 | | 0.137 | | 0.027 | | 0.485 | | -0.094 | | 0.009 | | 0.614 | | -0.133 | 0.101 | 0.412 | -0.071 | 0.006 | 0.506 |
| γ-GT (IU/L) | |  | |  | |  | |  | |  | |  | |  |  |  |  |  |  |
| Model 0 | | 0.194 | | 0.038 | | 0.272 | | -0.075 | | 0.006 | | 0.648 | | -0.151 | 0.023 | 0.339 | -0.105 | 0.011 | 0.313 |
| Model 1 | | 0.238 | | 0.039 | | 0.422 | | -0.347 | | 0.106 | | 0.103 | | -0.161 | 0.066 | 0.306 | -0.066 | 0.029 | 0.537 |
| Model 2 | | 0.199 | | 0.041 | | 0.268 | | -0.030 | | 0.012 | | 0.871 | | -0.148 | 0.081 | 0.340 | -0.104 | 0.011 | 0.318 |
| Model 3 | | 0.225 | | 0.044 | | 0.250 | | -0.082 | | 0.039 | | 0.653 | | -0.091 | 0.075 | 0.581 | -0.059 | 0.035 | 0.577 |
| Fatty liver index | |  | |  | |  | |  | |  | |  | |  |  |  |  |  |  |
| Model 0 | | 0.138 | | 0.019 | | 0.437 | | -0.048 | | 0.002 | | 0.771 | | -0.273 | 0.075 | 0.080 | 0.037 | 0.001 | 0.726 |
| Model 1 | | -0.076 | | 0.045 | | 0.797 | | -0.223 | | 0.044 | | 0.305 | | -0.289 | 0.185 | 0.053 | 0.073 | 0.012 | 0.512 |
| Model 2 | | 0.159 | | 0.063 | | 0.371 | | 0.063 | | 0.057 | | 0.732 | | -0.289 | 0.172 | 0.072 | 0.034 | 0.005 | 0.747 |
| Model 3 | | -0.358 | | 0.442 | | 0.148 | | 0.180 | | 0.195 | | 0.285 | | -0.102 | 0.522 | 0.390 | 0.091 | 0.188 | 0.354 |

P value provided for multiple regression analyses. β: standardized regression coefficient. Abbreviations: S-Klotho; shed form of the Klotho protein, HDL-C high-density lipoprotein cholesterol, LDL-C low-density lipoprotein cholesterol, ALT alanine transaminase, γ-GT; γ-glutamyl transferase.
